# Diversity of cervicovaginal human papillomavirus (HPV) genotypes and naturally occurring E6/E7 DNA polymorphisms of HPV-16 in Ghana

**DOI:** 10.1016/j.tvr.2023.200261

**Published:** 2023-05-11

**Authors:** Gladys Kaba, Andrew Stevenson, Samuel Asamoah Sakyi, Thomas Okpoti Konney, Ramya Bhatia, Nicholas A. Titiloye, Samuel A. Oppong, Francis Agyemang-Yeboah, Kate Cuschieri, Sheila V. Graham

**Affiliations:** a Department of Molecular Medicine, School of Medical Sciences, Kwame Nkrumah University of Science and Technology, Kumasi, Ghana; b Department of Biomedical Sciences, School of Basic and Biomedical Sciences, University of Health and Allied Sciences, Ho, Ghana; c Centre for Virus Research, School of Infection and Immunity, College of Medical, Veterinary and Life Sciences, Garscube Estate, University of Glasgow, Scotland, G61 1QH, UK; d Department of Obstetrics and Gynaecology, Komfo Anokye Teaching Hospital and Kwame Nkrumah University of Science and Technology, Kumasi, Ghana; e Scottish HPV Reference Laboratory, Department of Laboratory Medicine, Royal Infirmary of Edinburgh, University of Edinburgh, Scotland, EH16 4SA, UK; f Department of Pathology, School of Medical Sciences, Kwame Nkrumah University of Science and Technology Kumasi, Ghana; g Department of Obstetrics and Gynaecology, Korle-Bu Teaching Hospital and University of Ghana Medical School, Accra, Ghana

**Keywords:** Human papillomavirus, Genotyping, HPV E6/E7 DNA sequence Polymorphisms, Cervical cancer, HPV-16 variants

## Abstract

Human papillomavirus (HPV) E6 and E7 oncogene expression is essential for cervical carcinogenesis. Evidence exists that E6/E7 variants may have different transforming activities while the risk of HPV-16 variants (A/D) differs by race/ethnicity. We determined the type-specific diversity of HPV infection in women with high grade cervical disease or cervical cancer in Ghana and investigated naturally occurring E6/E7 DNA variants in this population. HPV genotyping was carried out on 207 cervical swab samples collected from women referred to a gynaecology clinic at two teaching hospitals in Ghana. HPV-16, HPV-18 and HPV-45 were detected in 41.9%, 23.3% and 16.3% of cases respectively. HPV-16 E6/E7 DNA sequencing was performed in 36 samples. Thirty samples contained E6/E7 variants of the HPV-16-B/C lineage. 21/36 samples were of the HPV-16C1 sublineage variant and all contained the E7 A647G(N29S) single nucleotide polymorphism (SNP). This study reveals the diversity of E6/E7 DNA and the dominance of HPV16 B/C variants in cervicovaginal HPV infection in Ghana. Type-specific HPV diversity analysis indicates that most Ghanaian cervical disease cases are vaccine preventable. The study provides an important baseline from which for the impact of vaccine and antivirals on clinically relevant HPV infection and associated disease can be measured.

## 1 Introduction

Globally about 570,000 cervical cancer (CxCa) cases and 311,365 associated deaths were recorded in 2018 [1,2]. More than 80% of CxCa cases were diagnosed in low and middle income countries (LMIC). In particular, in Africa in 2018 there was a total of 119,284 cases and 81,687 deaths [1].

In Ghana, out of a population of 30.8 million, 15.6 million are female. Next to cancer of the breast, cancer of the cervix is the second most prevalent female cancer with 3151 new cases and 2119 associated deaths recorded in 2018 [3]. Although Ghana's national policy on CxCa prevention, which is part of the National Reproductive Health Policy, was developed in 2005, it is yet to be implemented nationwide. Similarly, human papillomavirus (HPV) vaccination, which was expected to be an integral component of the CxCa control strategy is not yet implemented. In the absence of organised screening, there are avenues for opportunistic cervical screening in certain public and private health facilities depending on the individual's decision and/or requested by a healthcare provider [4,5]. As a result, women presenting to a healthcare facility with clinical suspicion of CxCa are referred for further assessment via colposcopy and/or biopsy for histology analysis.

HPV is the etiological agent of almost all (99.3%) cervical cancer cases globally [3,6,7] and the WHO strategy for elimination of cervical cancers includes primary prevention of HPV infection through vaccination in addition to HPV-based screening. Unfortunately, most LMIC countries are yet to implement the global strategy for the elimination of cervical cancer due to financial and political constraints and barriers to the introduction of the HPV vaccine [2,8].

E6 and E7 are the two main HPV oncoproteins and HPV-16 E6 and E7 gene sequence variants are associated with persistent HPV infection and progression to cancer [[9], [10], [11], [12]]. Among cervical precancers/cancers compared to controls, there are many sequence variants (single nucleotide polymorphisms: SNPs) within the E6/E7 gene region. For instance, lineage C and sublineages A4/D2/D3, have been documented to have a higher CIN3+ risk compared to sublineages A1/A2 [13]. HPV-16 variants may have diagnostic/treatment/management and immunoreactive implications [10,11,[13], [14], [15], [16], [17]].

Around 89% of cervical cancers are attributable to HPV-16/18/31/33/45/52/58 (de Martel et al., 2020). Although globally, HPV-16 is the predominant cancer-causing genotype, type-specific prevalence of medically important high risk HPVs (HR-HPVs) varies according to geographic area [17,18]. In Sub-Saharan Africa, the three most common HPV types detected in 74.5% of cervical cancers are HPV-16, HPV-18 and HPV-45 (in decreasing order of prevalence). While HPV types that are associated with cancers are broadly similar country to country, there is less information on variant distribution. Variants of an HPV genotype are defined by lineages and sublineages. For example, HPV-16 variants are classified into lineages (A/B/C/D) and sublineages (A1/A2/A3; B1/B2; C1/C2; D1/D2/D3). A number of HPV-16 A1/A2 variants (and some D variants) have been detected in most regions including North Africa but lineages B/C have been shown to be highly dominant in Sub-Saharan Africa [9,10]. However, these older studies do not represent contemporary variant profiles. Moreover, there is relatively limited data on variant-conferred risk of cervical cancer for the B/C lineages which are predominant in Africa [13].

The current study sought to determine the type-specific prevalence of cervicovaginal HPV genotypes in a contemporary cohort of cervical lesions and cervical cancers in Ghana. Moreover, we aimed to determine the common, clinically significant, naturally occurring cervicovaginal HPV-16 E6/E7 gene variants in this population. These data will set a baseline to help determine the nature of HPV-associated disease in Ghana and the potential impact of current and future vaccination strategies.

## 2 Methodology

### 2.1 Ethics

Ethical review and approval for the study was obtained from the Committee on Human Research Publication and Ethics (CHRPE), School of Medical Sciences, Kwame Nkrumah University of Science and Technology (KNUST-SMS)/Komfo Anokye Teaching Hospital, Kumasi (approval number-HRPE/AP/559/18) and Scientific Technical Committee/Institutional Review Board, Korle-Bu Teaching Hospital, Accra (approval number- KBTH-IRB/0081/2018).

### 2.2 Study design and study site

This cross-sectional study was conducted at the colposcopy service points and Combined Obstetrics and Gynaecology OPD/Family Planning Clinic, Obstetrics and Gynaecology Directorate, of Komfo Anokye Teaching Hospital, Kumasi and the cervical cancer treatment and/or management clinic, Department of Radiotherapy/Nuclear Medicine and the Family Planning Unit, Obstetrics and Gynaecology Department, Korle-Bu Teaching Hospital, Accra.

### 2.3 Study population

The study population included women with clinical suspicion of cancer of the cervix or confirmed diagnosis of CIN or CxCa who were referred for further assessment and/or treatment/management. Individuals without histology results at the time of sampling were primarily being referred for further assessment via colposcopy and/or biopsy to rule out CxCa. Those individuals already diagnosed with cervical precancer or cancer were recruited before the commencement of treatment. Histology or cytology analysis for diagnosis was made at laboratory centres and reported as part of routine clinical practice. All cervical cancer cases in this study were diagnosed via histology. All study participants presented with at least one gynaecological symptom associated with CxCa. Data collected included histology, age group (in years) 20–39, 40–59, 60–79, ≥80, HPV genotype profile (HPV positive/negative; one/more than one HPV type and specific HPV type).

### 2.4 Sampling, DNA extraction and HPV genotyping

Cervical swabs (cervicovaginal exfoliated cells) were collected from from 9^th^ October 2018 to 10^th^ November 2020 from consented individuals during routine medical examination. The cervical swab samples were preserved in a 20 ml solution (1 mM EDTA, 1 mM sodium citrate buffer pH 5 and 50% methanol) and stored at 4 °C. 209 out of 280 samples were available for HPV genotyping within the timeframe of the project. 10 ml aliquots of samples were transported at ambient temperature for centralized testing at the Scottish HPV Reference Laboratory (SHPVRL), in Edinburgh, for DNA extraction and HPV genotyping. Nucleic acid was extracted from 1 ml of the samples using the QIAamp Media MDx DNA extraction Kit (Qiagen) via the MDX platform and HPV infection in the samples was detected using the Optiplex HPV genotyping kit (a multiplex HPV genotyping assay by L1 DNA PCR/Luminex-detection) and following the manufacturer's instruction (Diamex, Heidelberg, Germany). Optiplex HPV genotyping is a Fluorescent Bead Assay (FBA) for diagnostic determination of 24 HPV types including the 12 established oncogenic high-risk (HR) HPV types (HPV-16/18/31/33/35/39/45/51/52/56/58/59), HPV-68 (probably carcinogenic HPV type), HPV-26/53/66/70/73/82 (possibly carcinogenic, HR-HPV types) and HPV-6/11/42/43/44 (low-risk (LR), HPV types). A random selection of HPV-16 L1 DNA positive samples were selected for E6/E7 DNA analysis (28 SCC, 2 ADC, 4 CIN2+, 2 undetermined pathology).

### 2.5 HPV-16 E6/E7 DNA PCR and analysis

Aliquots of DNA extracted from the samples were transported from the SHPVRL to the Centre for Virus Research, University of Glasgow, Scotland, where HPV-16 E6/E7 PCR was carried out on HPV-16 L1 DNA positive samples. PCR reactions used ACCUZYME™ Master Mix 2x (5 mM Mg^2+^, ultra-pure dNTPs, Accuzyme polymerase (high yield-high fidelity polymerase)) (Bioline-UK). Briefly, a total 25 μl PCR volume was used; DNA template; 1x ACCUZYME™ Master Mix (Bioline-UK), 0.2 μM HPV-16 E6/E7 forward (FW) primer; 0.2 μM reverse (RV) primer (Eurogentec-UK) (Supplementary Table 1) and nuclease-free water (Bioline-UK) was added to make up the total volume. 15 ng of DNA template was utilised in an initial PCR and where no PCR product was observed, the PCR was repeated for respective samples with 50 ng of DNA template. The HPV-16 PCR primers were designed to amplify the E6 and E7 open reading frames (ORFs) in one block. The annealing temperature was determined empirically by gradient PCR using DNA extract from W12GPXY cells, an HPV-16-positive invasive cervical cancer cell line [19]. The PCR cycling conditions were denaturation at 95 °C for 3 min, then 34 cycles of denaturation at 95 °C for 15s, annealing at 65 °C for 15s and, extension at 72 °C for 2 min. DNA from W12GPXY cells was used as the positive control for E6/E7 DNA amplification in every set of reaction [19]. The negative control was the reaction mix without addition of DNA. The PCR products were analysed by agarose gel electrophoresis. Where no product was observed after the first PCR reaction, a nested PCR was carried out on those samples using the primers as detailed in Supplementary Table 1.

### 2.6 HPV-16 E6/E7 DNA sequencing and analysis

PCR product purification and Sanger sequencing was carried out by Eurofins (UK). Two replicate sequencing reactions were carried out for each of the PCR-positive samples using forward and reverse sequencing primers as detailed in Supplementary Table 1. The resultant HPV-16 E6/E7 DNA sequence data was analysed using BLAST (https://blast.ncbi.nlm.nih.gov/Blast.cgi). Using the alignment report and printed text file, nucleotide variations observed were summarized in an excel spreadsheet. The classification of HPV-16 variants (lineages/sublineages) in the study was carried out according to the HPV-16 E6 lineage-specific diagnostic and non-lineage SNPs [9,20]. The SNPs observed were summarized with reference to previously reported HPV-16 genomes; (A1(K02718.1) HPV-16 reference; (A2-(AF536179), A3-(HQ644236.1), A4-(AF534061.1), B1-(AF536180.1), B2-(HQ644298.1), C-(AF472509.1), D1-(HQ644257.1), D2-(AY686579.1), D3-(AF402678.1)] [17].

### 2.7 Classification of HPV-16 variants

The classification of HPV-16 variants in the study samples was carried out as previously reported for lineage-specific diagnostic, likely sublineage diagnostic and non-lineage specific SNP patterns observed within the E6 gene region [[9], [20]]. Briefly, the HPV-16 B/C variants were distinguished from the other variants by presence of the five common E6 SNPs (C143G/G145T/T286A/A289G/C335T) which included the diagnostic SNP at C143G. Then among the classified B/C lineage samples, the previously reported likely sublineage variant diagnostic and/or associated non-lineage-specific SNPs were used to classify the B1/B2/C1 sublineages [B1(G132C; A83C), B2(A131G, T295G, T350G) and C1(T109C, G132T; A403G)] [[9], [10], [20]]. Any HPV-16 B/C classified variant in which the above likely sublineage-diagnostic B1/B2/C1 SNPs were not detected and having the same nucleotide as reference genome within the E6 gene, was classified as a C2 variant. Additionally, classified HPV-16 B/C variants detected with other SNPs, not previously utilised by Cornet et al., 2012 were not classified into sublineages.

## 3 Results

### 3.1 Sampling, demographics, clinical characteristics and HPV profile

**Fig. 1:**
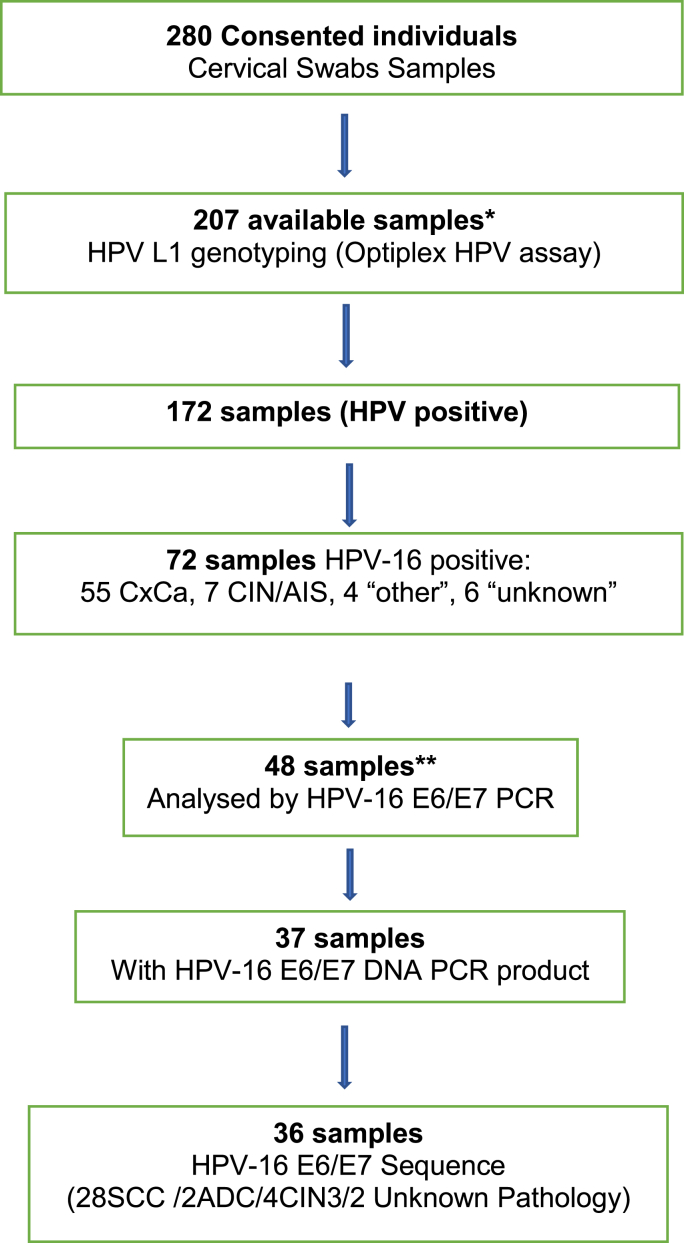
**Diagram of the study flow.** 208 sample were imported to the Scottish HPV Reference Laboratory and *207 samples gave HPV genotyping data. ** 48 HPV-16-positive samples were randomly selected for E6/E7 sequence analysis.

The study successfully recruited and collected cervical swab samples (cervicovaginal exfoliated cells) from 280 consented individuals recruited from 9^th^ October 2018 to 10^th^ November 2020 at two teaching hospitals in Ghana (Fig. 1). 209 out of 280 samples were available for subsequent molecular testing within the timeframe of the project in the UK. HPV genotyping was carried out on 207/209 samples as one sample was excluded because of missing data on age and another sample was excluded due to an operational error. Following routine histology or cytology practice in Ghana, CxCa was diagnosed in 74.9% (155/207) of samples, CIN in 8.2%, (17/207) samples, adenocarcinoma in situ (AIS) in 0.5% (1/207) samples, undetermined histology in 6.8% (14/207) samples and other histology findings in 9.6%, (20/207) of samples. The 155 CxCa cases included 89.7% (139/155 samples) squamous cell carcinoma (SCC), 8.4% (13/155 samples) adenocarcinoma (ADC), and 1.3% (3/155 samples) with “morphology not confirmed”. The age of the study participants at the time of sampling, ranged from 23 years to 85 years. The median age was 55 years and mean age was 56.1 years (SD ± 14.68). 80.2% (166/207) of the entire study population and 84.5% (131/155) of CxCa cases were within age range 40–79 years. Table 1 shows a summary of the 207 samples with data on histology, age and HPV profile.

**Table 1:** HPV status (HPV L1 DNA) and age group of the overall study population.

Histology	N (%)	Age Group (Years)				HPV Profile		No. of HPV type	
		20–39	40–59	60–79	>80	HPV-	HPV+	1HPV	>1HPV
SCC	139 (67.2)	8	69	49	13	14	125	84	41
ADC	13 (6.3)	1	5	5	2	5	8	6	2
CxCa Morphology not confirmed	3 (1.5)	0	2	1	0	1	2	1	1
CIN-1	7(3.4)	5	2	0	0	3	4	1	3
CIN-2	1(0.5)	1	0	0	0	1	0	0	0
CIN2/3	3(1.5)	1	2	0	0	0	3	3	0
CIN-3	6 (2.9)	0	3	3	0	0	6	5	1
AIS	1(0.48)	0	1	0	0	0	1	0	1
Other Histology	20 (9.7)	5	7	8	0	9	11	7	4
Undetermined Histology	14 (6.8)	4	2	7	1	2	12	7	5
Total	207 (100.0)	25	93	73	16	35	172	114	58

CxCa = Cervical Cancer. SCC=Squamous cell carcinoma. ADC = Adenocarcinoma. CIN= Cervical Intraepithelial Neoplasia. AIS = Adenocarcinoma In Situ.

Individuals with “Other histology” findings included.

2 Benign lesions - Cervical origin (Cervical Polyp/Cervical Ectropion).

4 Chronic inflammation – Cervical.

1 Previous Cervical Cancer.

1 Previous Cervical Cancer - Post Radiation.

1 Malignant lesion - non cervical origin (vagina).

2 Previous Cervical cancer coexisting with non-cervical cancer (vagina).

1 Benign lesion - non-cervical origin (vagina).

1 Benign lesion - non-cervical origin (endometrium).

4 Malignant lesion - non-cervical origin (endometrium).

1 Negative for Intraepithelial Lesion or Malignancy (NILM).

2 NILM- Previously HPV Positive.

### 3.2 The distribution of specific human papillomavirus genotypes

In total, 22 out of the 24 different HPV genotypes included in the genotyping assay were detected amongst the samples that were HPV-positive (Table 2). 172/207 samples (83.1%) were HPV-positive out of which 114/172 samples (66.3%) were positive for one HPV type whilst 58/172 samples (33.7%) were positive for more than one HPV type. 135/155 (87.1%) CxCa cases (125 SCC + 8 ADC+2 "morphology not confirmed" ) were HPV-positive (Supplementary Table 3). Thus 20/155 (12.9%) of CxCa cases were HPV-negative including 14 SCC, 5 ADC, and one CxCa designated “morphology not confirmed”.

**Table 2:** Frequency Distribution of Specific HPV Types and Histology categories positive for one HPV type or more than one HPV type.

HPV TYPE	Cervical Cancer	Precancer (Cervix)	Other Histology	Undetermined Histology
	aN = 197	bN = 22	cN = 18	dN = 17
	N (%)	N (%)	N (%)	N (%)
HPV-16	55 (27.92)	7 (31.82)	4 (22.22)	6 (35.29)
HPV-18	36 (18.27)	2 (9.09)	1 (5.56)	1 (5.88)
HPV-45	21 (10.66)	2 (9.09)	3 (16.67)	2 (11.76)
HPV-35	13 (6.60)	1 (4.55)	2 (11.11)	1 (5.88)
HPV-52	14 (7.11)	2 (9.09)	0 (0.00)	1 (5.88)
HPV-42	8 (4.06)	2 (9.09)	0 (0.00)	1 (5.88)
HPV-58	8 (4.06)	0 (0.00)	1 (5.56)	1 (5.88)
HPV-56	6 (3.05)	0 (0.00)	2 (11.11)	0 (0.00)
HPV-31	6 (3.05)	0 (0.00)	0 (0.00)	0 (0.00)
HPV-66	4 (2.03)	0 (0.00)	2 (11.11)	0 (0.00)
HPV-70	3 (1.52)	1 (4.55)	1 (5.56)	1 (5.88)
HPV-53	3 (1.52)	1 (4.55)	0 (0.00)	1 (5.88)
HPV-59	2 (1.02)	1 (4.55)	0 (0.00)	1 (5.88)
HPV-82	4 (2.03)	0 (0.00)	0 (0.00)	0 (0.00)
HPV-33	0 (0.00)	2 (9.09)	0 (0.00)	1 (5.88)
HPV-39	3 (1.52)	0 (0.00)	0 (0.00)	0 (0.00)
HPV-51	3 (1.52)	0 (0.00)	0 (0.00)	0 (0.00)
HPV-68	1 (0.51)	1 (4.55)	1 (5.56)	0 (0.00)
HPV-43	1 (0.51)	0 (0.00)	1 (5.56)	0 (0.00)
HPV-44	2 (1.02)	0 (0.00)	0 (0.00)	0 (0.00)
HPV-6	2 (1.02)	0 (0.00)	0 (0.00)	0 (0.00)
HPV-73	2 (1.02)	0 (0.00)	0 (0.00)	0 (0.00)
**TOTAL**	**197 (100.00)**	**22 (100.00)**	**18 (100.00)**	**17 (100.00)**

a N = The total number of specific HPV types detected in cervical cancer cases that were HPV-positive.

b N = The total number of specific HPV types detected in precancer cases that were HPV-positive.

c N = The total number of specific HPV types detected in samples documented as other histology findings that were HPV-positive.

d N = The total number of specific HPV types detected in documented as unknown histology that were HPV-positive. Percentages were calculated using the total number of all specific HPV types detected in respective histology categories positive for one or more than HPV type (1 or >1HPV type) as the denominator. The total number of a specific HPV type detected among samples positive for one or more than one HPV type (1 or >1HPV type) was used as the numerator.

The three most common HPV types (HPV-16/18/45) were detected in 70 (76.9%) of the 91 CxCa cases positive for a single HPV genotype. HPV-16 alone was found in 39/91 cases (42.7%) and with other HPV genotypes in 55/135 cases (40.7%). HPV-18 was present alone in 19/91 (20.9%) of cases or with other genotypes in 36/135 (26.7%) of cases while HPV-45 was found alone in 12/91 (13.2%), or with other genotypes in 21/135 (15.6%) of cases. One SCC samples was positive for a single HPV-73 (Possibly Carcinogenic HR-HPV Type) and another for HPV-73/82 (possibly carcinogenic HR-HPV Types).

**Fig. 2:**
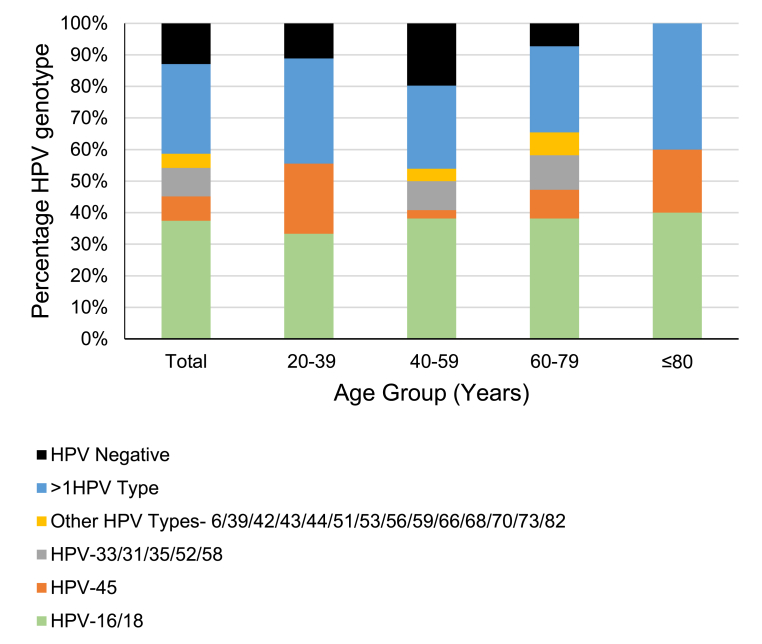
**Graph of age versus HPV genotypes among cervical cancer (CxCa) cases (N=155)**. Age groups are as shown in Table 1. HPV-16 and HPV-18 are grouped as the two genotypes causing the greatest burden of cervical cancers (green bars). HPV-45 is grouped alone due to its dominance in African populations (orange bars). Prevalent HPV genotypes 31, 33, 35, 52 and 58 are in a third group (gray bars). Samples positive for more than one HPV type (blue bars) included coinfection with HPV-16 and/or HPV-18 and/or HPV-45 or other HPV type(s). No HPV genotype was counted twice. The numbers in the Figure add up to 100%. The remaining HPV genotypes detectable using the Optiplex genotyping assay are in a fourth group (yellow bars). HPV-negative cases are shown with black bars. (For interpretation of the references to colour in this figure legend, the reader is referred to the Web version of this article.)

For this study population, the majority were in the 40–59 year age range, four were aged between 60 and 79 years and one was in the age range 20–39 years. The average age was 51 years. Fig. 2 shows the HPV genotype distribution across the age ranges. All 15 CxCa samples from individuals ≥80 years in this study were HPV-positive. The 20–39 years and the ≥80 years age groups had the highest percentage of >1 HPV genotype and there was a higher percentage of HPV-45 detection.

77.8% (14/18) of CIN/AIS cases were HPV-positive. Nine were positive for one HPV type and five for more than one HPV type. 55% (11/20 samples) of cases of “other” histology (Table 1), which ranged from benign polyps to malignant lesions of non-cervical origin, were HPV-positive. Seven were positive for a single HPV type and four for more than one HPV type.

### 3.3 HPV-16 E6 DNA sequence polymorphism

HPV-16 genome variants, including SNPs within HPV oncogenes E6/E7, and the inherent risk associated with some variants (lineages-A/D) differ by race/ethnicity, but African studies are limited. Thus, E6/E7 PCR was performed on a random selection of 48 of the 72 samples (Supplementary Table 2) that were positive for HPV-16. Using a standard one-step PCR, products were amplified from 21 samples. Nested PCR was able to detect E6/E7 DNA in a further 16 samples. Of the 37 samples, 36 yielded adequate sequence information for analysis. SNPs observed in the PCR-amplified region are summarized in Table 3 and shown in comparison with reference HPV-16 variant genomes (see Supplementary Fig. 1). Only 4/36 of the nucleotide sequence had E6 DNA sequence data that was similar to the reference HPV-16 genome (A1 = K02718.1). The five common E6 SNPs (*C143G/G145T/T286A/A289G/C335T*) specific for HPV-16 B/C variants were observed in 32/36 (88.89%) samples. E6 SNPs specific for HPV-16 B1/C1 sublineages (including the C1 non-lineage specific SNP *T109C*) were present in 26 out of these 32 samples. Specifically, 20/26 samples had C1 SNPs (*T109C/G132T/403G*) and 6/26 samples had B1 SNPs (*A83C/G132C*). Additionally, two samples were classified as HPV-16C2. The remaining four samples mapped to the HPV-16 B/C lineage variant but could not be classified into sublineages. Two of these contained E6 SNP *A131* but nucleotides were unchanged at *T295* or *T350* with respect to HPV-16 reference genome A1. They are likely diagnostic for the B2 lineage [17]. In a further two samples the DNA sequence data was equivocal at key SNPs but these samples were most likely to be B1 and C1 respectively based on the presence of other SNPs representative of these sublineages.

**Table 3:**
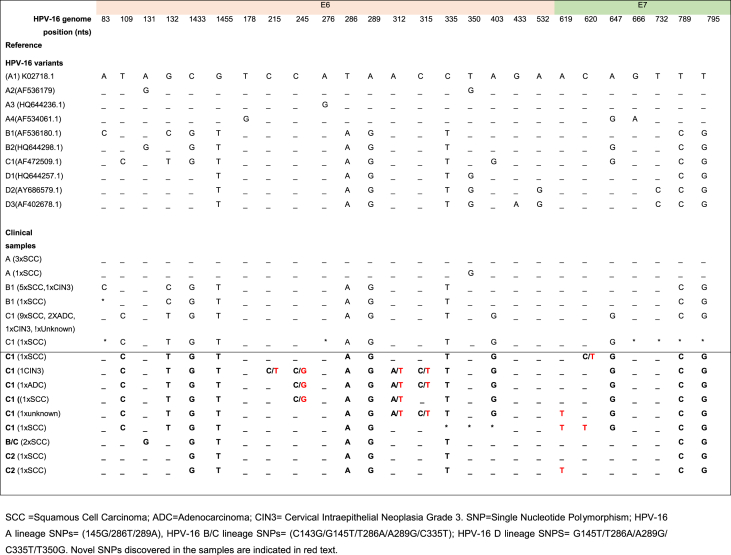
Single nucleotide polymorphisms observed in HPV-16 E6/E7 DNA sequence, among 36 women, summarized alongside previously reported HPV-16 variant genomes.

### 3.4 HPV-16 E7 DNA sequence polymorphism

Only 4/36 of the studied samples had an E7 DNA sequence that was similar to the reference HPV-16 genome (A1: K02718.1) (Table 3). The HPV-16 E7 DNA SNPs (T789C/T795G) were observed in the 32/36 samples, which were classified as B/C variants by E6 DNA sequence information as described above. E7 SNP A647G(N29S) was detected in 21/36 samples. All E7 SNPs were detected together with E6 SNPs G132T/403G (likely diagnostic) and T109C (previously reported) for HPV-16 sublineage C1.

### 3.5 Dual nucleotide changes at one nucleotide position in the same sample

In 6/36 HPV-16-positive samples, dual nucleotide changes were observed in the same sample at different nucleotide positions. Apart from C215 C/T, all observed dual changes included one nucleotide which was unchanged from the HPV-16 reference genome (A1). The other nucleotide changes appear to be novel SNP candidate (that is C245**G**; A312**T**; C315**T**; C620**T**). Furthermore, another novel SNP candidate, A619**T**, was also detected in three samples (Table 4A). Lone SNPs not observed in any of the reference HPV-16 genomes were detected in 11/36 samples (Table 4B). Three of these were reported previously but the remainder are reported here for the first time.

**Table 4A:**
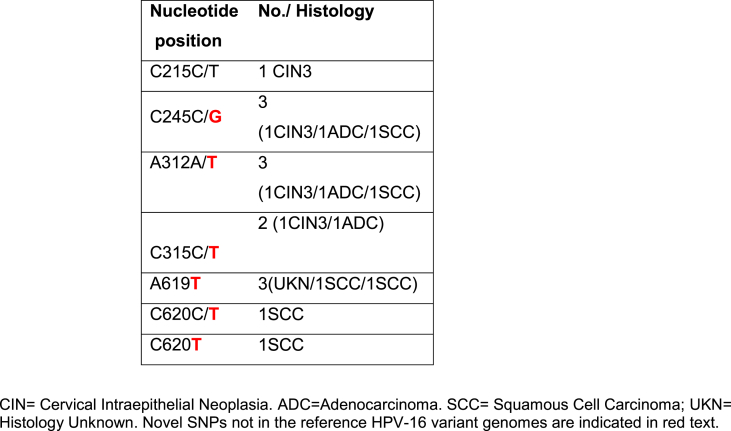
Dual nucleotide changes at one nucleotide position in the same sample, or SNPs observe in more than one sample and not in the reference HPV-16 variant genomes.

**Table 4B:** E6/E7 nucleotide changes that occurred in a single sample that are not in the reference HPV-16 variant genomes.

SNP	Reference
∗A 92 A/C	
A 167 A/T	
∗A 174 A/C	
∗G 176 G/A	[14,38,39]
G 188 G/A	[38] but in some lineage-A variants and G188C in some lineage-A/B variants
G 269 A	[38] but in some lineage-A variants
G 545 G/A	
T 228 T/A; C 627C/T	
A 768 A/T; A 770 A/T	

∗ = HPV-16-A-lineage variant.

## 4 Discussion

In this study we determined the HPV genotype and E6/E7 variant landscape in a highly disease-enriched population in Ghana. This means that we have preferentially assessed variants with transforming activity rather than performing a mapping exercise of variant prevalence in the (largely disease-free) general population. In the absence of such a control population we cannot make statements on viral fitness. Thus, this cross-sectional study was carried out to determine the diversity of cervicovaginal HPV genotypes and characterise the naturally occurring HPV-16 E6/E7 DNA SNPs (and lineage/sublineage variants) in individuals with clinical suspicion of cancer of the cervix or diagnosed with CxCa or precancer of the cervix. HPV genotyping indicated 83% of samples were HPV-positive. Of the cervical cancers, all were positive for at least one high-risk type, with 67.4% being positive for type 16 and/or 18.

Most of the cervical cancer cases were squamous cell carcinoma (SCC) with a lower percentage of adenocarcinoma (ADC) giving an ADC to SCC ratio of about 1:11, which is typically observed in LMIC that are yet to implement nationwide, organised cervical cancer screening programmes. This ADC to SCC ratio is consistent with previous Ghanaian studies by Denny et al. (2014) who reported a ratio of 1:10 (15/155 samples, 9.7%) and Awua et al. (2016) who reported a ratio of 1:12 (19/246 samples, 7.7%). A worldwide study reported an ADC to SCC ratio of 1:18 (5.3%) [21] while studies carried out in developed countries (such as Australia and the US) showed a higher ratio of about 1:3 [22,23].

87.1% of CxCa cases were HPV-positive. This is similar to some worldwide studies which reported 85%–89.9% positivity [24,25]. HPV positivity in this study was based on HPV L1 DNA genotyping in cervical swab samples (exfoliated cells). In another Ghanaian study using HPV type-specific L1 DNA based testing in FFPE samples HPV was detected in 93.9% CxCa cases [26] whereas a third Ghanaian study using HPV E6 genotyping of archival FFPE samples reported 89.8% HPV positivity [27]. Thus, this study agrees with previous estimates.

As a single genotyping test was employed in this study and given that some cervical cancers are associated with an HPV-independent pathway to transformation, some HPV-negative CxCa cases were expected. We acknowledge that some of the HPV-negative CxCa cases in this study may represent false negatives due to HPV genome integration leading to a lack of detection by L1 PCR. A reassessment of the HPV L1-negative CxCa samples by targeting other HPV genome region(s) including E6/E7, and through performing deep whole genome sequencing, is likely to increase the percentage occurrence of HPV-positivity in the CxCa cases reported in this study.

In the current study, the three most common HPV types detected in CxCa cases positive for a single HPV genotype were HPV-16 (42.9%), −18 (20.9%), and −45 (13.2%). These genotypes are the most predominant HPV types in CxCa cases globally (∼74.5%), including in Africa. In West Africa, the percentage occurrence of HPV-16/18/45 in CxCa cases was previously reported to be about 70.1%; HPV-16 (35.5%), HPV-18 (20.1%), HPV-45 (16.5%) [3,28]. In a previous study, Denny et al. also observed HPV-16, HPV-18, and HPV-45 as the most prevalent genotypes in CxCas diagnosed in Ghana. However, Awua et al. (2016) reported HPV-18, HPV-59, HPV-45 in decreasing order of prevalence, as the three most common HPV types. In Ghana, where the quadrivalent HPV vaccine has been introduced in a pilot program, estimates are that it will protect up to around 60% of women from HPV infection/CxCa. Our study shows that up to around 60% of CxCa cases could have been prevented by either the bivalent or quadrivalent HPV vaccines while, an estimate of >90% of CxCa cases could be prevented by the nonavalent HPV vaccine (Supplementary Table 3). However future HPV vaccines in Ghana should also be protective against HPV-45. Hence introduction of the bivalent HPV vaccine in Ghana may be beneficial for a nationwide vaccination programme due to its reported cross protection against HPV-45.

This study reported a high prevalence of HPV-16 in high grade lesions and cancer in Ghana, with comparable prevalence to the work of Denny et al., 2014 [26], however a report by Awua et al. (2016) [27] showed a lower prevalence of HPV-16. Differences in sensitivity of HPV genotyping assays (L1 DNA versus E6 DNA testing) and/or archival FFPE tissues versus freshly collected cervical swab samples may in part be responsible for these differences, as may the time frame of the studies and recruitment criteria. It is also feasible that the distribution of E6 SNPs in Africa may have impacted on the performance of the PCR-E6-primer set utilised for HPV-16 testing among CxCa cases in Ghana in the study by Awua et al., 2016. Further investigation is however warranted.

Our data is observational but adds to current understanding of HPV E6/E7 variant prevalence and distribution in Africa. Viral oncogene variants have been linked with progression to cancer. This study provides preliminary data in Ghana on E6/E7 DNA sequence polymorphism of HPV-16 within a highly-disease enriched population. The data can serve as the basis for epidemiological studies to ascertain the spread and impact of these HPV type-specific variants in the Ghanaian population, which may be important for screening, prevention, and treatment/management decisions. In future, we would like to complement the work to ascertain the pattern of variants in the healthy population. HPV-16 variants are population specific, and the B/C lineages are dominant in Sub-Saharan Africa [9,10,27,[29], [30], [31], [32], [33]]. Most Ghanaian samples were of the HPV-16 B/C variant and only 11% were classified as the A lineage, which was been reported to occur in North African populations (Cornet et al., 2013). Of the few (19%) HPV-16 B lineage variants, all were of B1 sublineage. We found the HPV-16C lineage to be dominant (64%) with high percentage occurrence of sublineage C1 (58%) in agreement with Cornet et al., 2012 who reported previously that HPV-16 lineage C was equally distributed in North Africa (Algeria and Morocco) and Sub-Saharan Africa (Benin/Guinea/Kenya/Mali/Nigeria/South Africa/Tanzania/Uganda) [9].

Our study also confirms B/C lineages to have the E7 SNPs (T789C/T795G) previously reported as non-lineage specific as they are also observed in the A and D lineages [14,34]. The E7 variants with the SNP A647G(N29S) were however detected in all samples classified as HPV-16C1 by the E6 SNPs (T109C/G132T/A403G). The A647G (N29S) E7 SNP has been observed previously in some cervical cancer cases from Tanzania [35], and has been detected in HPV-16 A/B/C variants making it non-lineage specific [14,34,36]. This E7 variant has been biochemically described in vitro to have an increased oncogenicity [11] but cervical cancer risk association is conflicting across the various studies. These findings highlight the need to ascertain the spread of the E7 A647G (N29S) covariation with E6 SNPs (109C/132T/403G) among cervical cancer cases and the cervical cancer risk of the E7 A647G(N29S) SNP, especially among HPV-16C lineage variants in Ghana.

A minority of the study samples (6/36 samples) positive for a single HPV type (HPV-16) showed dual nucleotide changes in a single position possibly due to the presence of multiple HPV-16 variants in the same sample [12,37]. Therefore, assessment of the relevant samples by next-generation deep sequencing methodology for the confirmation of minor variants in the studied samples could be insightful [37]. Among the following SNPs C245 C/**G**; A312 A/**T**; C315 C/**T**; C620 C/**T** with dual nucleotide changes observed this study, only one of the nucleotide changes (un-bolded text) was unchanged as in the HPV-16 reference genome (K02718.1) and/or previously reported [9,14,17,20,38], whilst the other nucleotide changes (bold text) appeared to be candidate novel SNPs and were detected in more than 2 samples. Another SNP that may be considered novel, A619**T**, was also detected in 3 samples. Thus, five SNPs (C245**G**/312**T**/315**T**/A619**T**/C620**T**) detected in two or more samples are being proposed as naturally occurring, candidate novel SNPs [9,14,17,20,30,31,34,38,39].

In this study histology was carried out at centres providing routine pathology services in Ghana and a consensus review of histology was not performed as a consequence of this research. We accept that a consensus opinion would have made histology data more robust technically (particularly for the CIN cases). However, we were also keen to deliver a study that reflected the HPV prevalence in routinely diagnosed high grade lesions and cancer in Ghana. Our study is also limited by sample size, especially considering the limited number of samples positive for the HPV-16-B lineage variant. However, the cases were sampled from the two major teaching hospitals in Ghana. Therefore, the distribution of HPV-16 variants we observed among cervical tumours may reflect the wider distribution in the Ghanaian population. Although eleven out of the 48 samples gave no E6/E7 PCR product, all were positive by β-globin and GAPDH control PCR analysis. Factors such as the presence of other HPV genotypes, loss of HPV E6/E7 DNA and/or low viral copy number, might have impacted the PCR outcome. Nonetheless, it would be interesting for future work in Ghana to look at case-control analysis of the spread and impact of HPV variants in Ghana, especially HPV-16-sublineage-C1, with E6 SNPs (109C/132T/403G) detected together with E7 SNP 647G (N29S).

### 4.1 Conclusions

This study confirms the dominance of the HPV types-16/18/45 with a high percentage occurrence of single HPV-16 infection in cervical cancer cases in Ghana using L1 DNA genotyping. The study provides the first dataset describing the naturally occurring HPV-16 E6/E7 DNA sequence variance in cervix cancer/precancer cases in Ghana and confirms the dominance of the B/C lineage variant and the rarity of the A lineage in Africa. The study documents the dominance of the HPV-16 C1 lineage variant and the presence of the E7 647G (N29S) SNP in all our C1 lineage cases. Characterisation of E6/E7 DNA sequence variants can be very important when newer treatment/management of cervical cancer cases, that involves HPV-16 E6 and/or E7 is to be considered in the future, in particular if therapeutic E6/E7 vaccines become available. Our data will also serve as a contemporary baseline for future trials where the impact of prophylactic and therapeutic vaccine and HPV antivirals are measured.

## Funding

GK was a recipient of a Commonwealth Split-Site Scholarship grant number GHCN201823 and also received a Ghana Public Universities Senior Members Book and Research allowance for research activities carried out in Ghana.

## Author contributions

GK: Data curation. Visualisation. Formal analysis. Funding acquisition. Writing – original draft. AS: Data curation, methodology, resources. SAS: Supervision. Writing – original draft review and editing. TOK: Supervision (KATH), Project administration. RB: Data curation. NAT: Conceptualization, Supervision. SAO: Supervision (KBTH), Project administration. FA-Y: Conceptualization, Supervision. KC: Conceptualization. Funding acquisition. Supervision. Writing – review and editing. SVG: Conceptualization. Funding acquisition. Supervision. Project administration. Writing –original draft, review and editing.

## Declaration of competing interest

The authors declare that they have no known competing financial interests or personal relationships that could have appeared to influence the work reported in this paper.

## Data Availability

Data will be made available on request.
