# Supplementary material for: Diversity of cervicovaginal human papillomavirus (HPV) genotypes and naturally occurring E6/E7 DNA polymorphisms of HPV-16 in Ghana
Source: Tumour Virus Res. 2023 May 11;15:200261. doi: 10.1016/j.tvr.2023.200261 (PMC10209332; doi:10.1016/j.tvr.2023.200261)
Supplement: Multimedia component 1 [file mmc1.docx]

## Supplementary Table 1: Primer sequences for the PCR amplification and sequencing of the HPV-16 E6-E7 open reading frames.

| **Primer name** | **Primer Sequence** | **Primer position on the HPV16 genome (nts)** | **Expected product size (bps)** |
| --- | --- | --- | --- |
| E6 forward primer | 5’-CACATGGGTGTGTGCAAACCG-3’ | 7840-7860 | 1007bp |
| E7 reverse primer | 5’-TTCCACTACAGCCTCTAC-3’ | 942-926 |  |
| Nested E6 forward primer | 5’-GGGCGTAACCGAAATCGG-3’ | 28-45 | 855bp |
| Nested E7 reverse primer | 5’-ACCTGCAGGATCAGCCATGG-3’ | 882-863 |  |
| Forward sequencing primer | 5’-CGGTTGAACCGAAACCGGTTAG-3’ | 43-64 | 833bp |
| Reverse sequencing primer | 5’-GGATCAGCCATGGTAGATTATGG-3’ | 875-853 |  |
| GAPDH forward  primer | 5’-CGCTCTCTGCTCCTCCTGTT-3’ | N/A | 435bp |
| GAPDH reverse  primer | 5’-CCATGGTGTCTGAGCGATGT-3’ | N/A |  |

**Supplementary Table 2. A Summary Table on The Histology, HPV Status and Age Group of Samples Positive For HPV-16**

|  | **N(%)** |  | **Age Group (Years)** | | | |  | **HPV Type(s)** | |
| --- | --- | --- | --- | --- | --- | --- | --- | --- | --- |
| **Histology** |  |  | 20-39 | 40-59 | 60-79 | 80 and above |  | 1HPV | >1HPV |
| Squamous cell carcinoma | 53(73.61) |  | 4 | 23 | 21 | 5 |  | 37 | 16 |
| Adenocarcinoma | 2(2.77) |  | 0 | 1 | 1 | 0 |  | 2 | 0 |
| CIN3 | 5(6.94) |  | 0 | 3 | 2 | 0 |  | 4 | 1 |
| CIN2/3 | 1(1.38) |  | 0 | 1 | 0 | 0 |  | 1 | 0 |
| CIN1 | 1(1.38) |  | 1 | 0 | 0 | 0 |  | 0 | 1 |
| Unknown histology | 6(8.33) |  | 2 | 1 | 3 | 0 |  | 4 | 2 |
| Chronic inflammation – Cervical | 1(1.38) |  | 0 | 1 | 0 | 0 |  | 1 | 0 |
| Benign lesion - Cervical origin  (non-tumour) | 1(1.38) |  | 1 | 0 | 0 | 0 |  | 1 | 0 |
| NILM- Previous HPV Positive | 1(1.38) |  | 1 | 0 | 0 | 0 |  | 0 | 1 |
| Benign lesion-non cervical origin  (Endometrium) | 1(1.38) |  | 0 | 1 | 0 | 0 |  | 1 | 0 |
|  | **72(100.00)** |  | **9(12.5%)** | **31(43.1%)** | **27(37.5%)** | **5(6.9%)** |  | **51(70.8%)** | **21(29.2%)** |

CIN 1/2/3=Cervical Intraepithelial Neoplasm Grade 1 or 2 or 3; NILM= Negative for Intraepithelial Lesion or Malignancy

**Supplementary Table 3: Specific HPV Type Burden Among CxCa Cases (1HPV or 1HPV/ >1HPV Type)**

|  |  |  | Minimum Estimate | |  |  | Any-Type Estimate | |
| --- | --- | --- | --- | --- | --- | --- | --- | --- |
| HPV Type | 1HPV |  |  |  |  | 1HPV/  >1HPV |  |  |
|  | N | 1N =91 | 2N =135 | 3N =155 |  | N | 2N =135 | 3N =155 |
|  |  | % | % (95% CI) | % (95% CI) |  |  | % (95% CI) | % (95% CI) |
| HPV-16 | 39 | 42.9 | 28.9 (21.2, 37.3) | 25.2 (18.5, 32.7) |  | 55 | 40.7 (32.4, 49.5) | 35.5 (28.0, 43.6) |
| HPV-18 | 19 | 20.9 | 14.1 (8.7, 21.1) | 12.3 (7.5, 18.5) |  | 36 | 26.7 (19.4, 35.0) | 23.2 (16.8, 39.7) |
| HPV-45 | 12 | 13.2 | 8.9 (4.7, 15.0) | 7.7 (4.1, 13.1) |  | 21 | 15.6 (9.9, 22.8) | 13.6 (8.6, 20.0) |
| HPV-35 | 6 | 6.6 | 4.4 (1.7, 9.4) | 3.9 (1.4, 8.2) |  | 13 | 9.6 (5.2, 15.9) | 8.4 (4.5, 13.9) |
| HPV-58 | 5 | 5.5 | 3.7 (1.2, 8.4) | 3.2 (1.1, 7.4) |  | 8 | 5.93 (2.6, 11.3) | 5.2 (2.3, 9.9) |
| HPV-52 | 2 | 2.2 | 1.5 (0.2, 5.3) | 1.3 (0.2, 4.5) |  | 14 | 10.4 (5.8, 16.8) | 9.0 (5.0, 14.7) |
| HPV-39 | 3 | 3.3 | 2.2 (0.5, 6.4) | 1.9 (0.4, 5.6) |  | 3 | 2.2 (0.5, 6.4) | 1.9 (0.4, 5.6) |
| HPV-51 | 2 | 2.2 | 1.5 (0.2, 5.3) | 1.3 (0.2, 4.6) |  | 3 | 2.2 (0.5, 6.4) | 1.9 (0.4, 5.6) |
| HPV-59 | 1 | 1.1 | 0.7 (0.0, 4.1) | 0.7 (0.0, 3.5) |  | 2 | 1.5 (0.2, 5.3) | 1.3 (0.2, 4.6) |
| HPV-73 | 1 | 1.1 | 0.7 (0.0, 4.1) | 0.7 (0.0, 3.5) |  | 2 | 1.48 (0.2, 5.3) | 1.3 (0.2, 4.6) |
| HPV-31 | 1 | 1.1 | 0.7 (0.0, 4.1) | 0.7 (0.0, 3.5) |  | 6 | 4.4 (1.6, 9.4) | 3.9 (1.4, 8.2) |
| HPV-42 | 0 | 0 | 0 | 0 |  | 8 | 5.9 (2.6, 11.3) | 5.2 (2.3, 9.9) |
| HPV-56 | 0 | 0 | 0 | 0 |  | 6 | 4.4 (1.7, 9.4) | 3.9 (1.4, 8.2) |
| HPV-66 | 0 | 0 | 0 | 0 |  | 4 | 3.0 (0.8, 7.4) | 2.6 (0.7, 6.5) |
| HPV-70 | 0 | 0 | 0 | 0 |  | 3 | 2.2 (0.5, 6.4) | 1.9 (0.4, 5.6) |
| HPV-53 | 0 | 0 | 0 | 0 |  | 3 | 2.2 (0.5, 6.4) | 1.9 (0.4, 5.6) |
| HPV-82 | 0 | 0 | 0 | 0 |  | 4 | 3.0 (0.8, 7.4) | 2.6 (0.7, 6.5) |
| HPV-68 | 0 | 0 | 0 | 0 |  | 1 | 0.7 (0.0, 4.1) | 0.7 (0.0, 3.5) |
| HPV-43 | 0 | 0 | 0 | 0 |  | 1 | 0.7 (0.0, 4.1) | 0.7 (0.0, 3.5) |
| HPV-44 | 0 | 0 | 0 | 0 |  | 2 | 1.5 (0.2, 5.3) | 1.3 (0.2, 4.6) |
| HPV-6 | 0 | 0 | 0 | 0 |  | 2 | 1.5 (0.2, 5.3) | 1.3 (0.2, 4.6) |
| TOTAL | 91 | 100 | 67.4 | 58.7 |  | 197 | 145.9 | 1127.1 |
|  |  |  |  |  |  |  |  |  |
|  | N | % | % (95% CI) | % (95% CI) |  |  | % (95% CI) | % (95% CI) |
| HPV-16/18/45 | 70 | 76.9 | 51.9 (43.1, 60.5) | 45.2 (37.2, 53.4) |  | 112 | 83.0 (75.5, 88.9) | 72.3 (64.5, 79.1) |
| Bivalent Vaccine HPVs | 58 | 63.7 | 43.0 (34.5, 51.8) | 37.4 (29.8, 45.5) |  | 91 | 67.4 (58.8, 75.2) | 58.7 (50.5, 66.6) |
| Quadrivalent Vaccine HPVs | 58 | 63.7 | 43.0 (34.5, 51.8) | 37.4 (29.8, 45.5) |  | 93 | 68.9 (60.4, 76.6) | 60.0 (51.8, 67.8) |
| 9-valent Vaccine HPVs | 78 | 85.7 | 57.8 (49.0, 66.2) | 50.3 (42.2, 58.4) |  | 142 | 105.2 | 91.6 (86.1, 95.5) |
| Vaccine HR HPVs | 78 | 85.7 | 57.8 (49.0, 66.2) | 50.3 (42.2, 58.4) |  | 140 | 103.7 | 90.3 (84.5, 94.5) |
| Non-vaccine HR HPVs | 12 | 13.2 | 8.9 (4.7, 15.0) | 7.7 (4.1, 13.1) |  | 27 | 20 (13.6, 27.8) | 17.4 (11.8, 24.3) |
| HR HPVs | 90 | 98.9 | 66.7 (58.0, 74.5) | 58.1 (49.9, 65.9) |  | 167 | 123.7 | 107.7 |

Percentages were calculated using the total number of specific HPV types detected in respective histology categories positive for one HPV type as the denominator^. 1^N = Total number of specific HPV types among cervical cancer cases^. 2^N = Total number of specific HPV types among cervical precancer cases^. 3^N = Total number of specific HPV types among individual with other histology^. 4^N = Total number of specific HPV types among individuals with unknown histology.

A


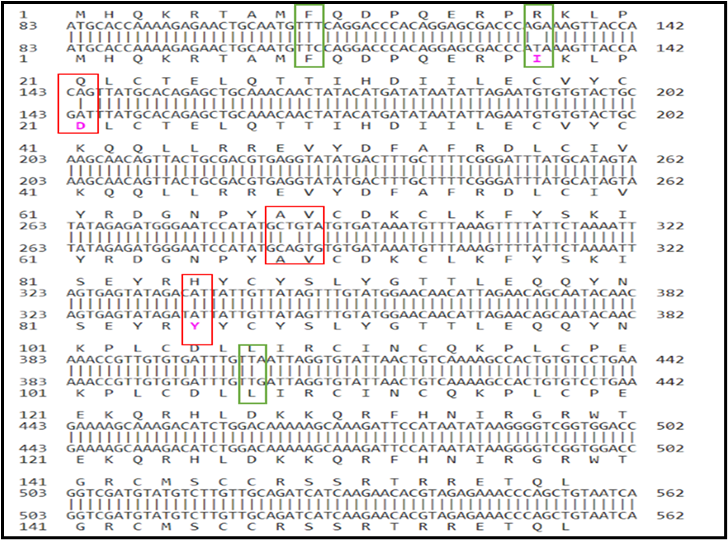


B


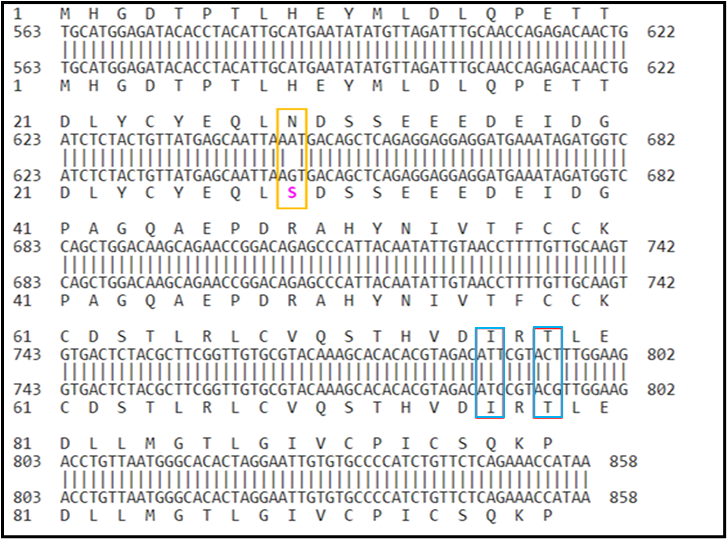


**Supplementary Figure 1.** Nucleotide sequence and corresponding amino acid sequence of the HPV-16 E6/E7 gene region. (A) E6 gene region. Highlighted in red are the E6 SNPs common to HPV-16 lineage B/C) including a diagnostic SNP (C143G). Highlighted in green are HPV-16 sublineage C1 likely diagnostic E6 SNPs (G132T; A403G) and a non-lineage specific SNP that has been observed in sublineage C1 (T109C), highted in black are E6 splice donor splice site (nt 226) and acceptor splice sites (nt 409 and nt 526); (B) E7 gene region. Highlighted in blue are the non-lineage specific HPV-16 E7 SNPs at nucleotide common to the B/C lineage variant; Highlighted in orange is a non-lineage specific HPV-16 E7 SNP A647G
